# Capturing the Electrical Activity of all Cortical Neurons: Are Solutions Within Reach?

**DOI:** 10.1002/advs.202506225

**Published:** 2025-06-27

**Authors:** Attila Kaszás, Domokos Meszéna, Richárd Fiáth, Andrea Slézia, István Ulbert, Gergely Katona

**Affiliations:** ^1^ Institute of Cognitive Neuroscience and Psychology HUN‐REN Research Centre for Natural Sciences Budapest 1117 Hungary; ^2^ Mines Saint‐Etienne Centre CMP, Département BEL Gardanne F‐13541 France; ^3^ Institut de Neurosciences de la Timone CNRS UMR 7289 & Aix‐Marseille Université Marseille 13005 France; ^4^ Center for Neurotechnology and Neurorecovery Department of Neurology Massachusetts General Hospital Harvard Medical School Boston MA 02114 USA; ^5^ Faculty of Information Technology and Bionics Pázmány Péter Catholic University Budapest 1083 Hungary; ^6^ Department of Neurosurgery and Neurointervention Faculty of Medicine Semmelweis University Budapest 1145 Hungary

**Keywords:** brain‐computer interface, foreign body response, high‐density recording, intracortical electrode, neural interface

## Abstract

Recent advancements in high‐density implantable intracortical electrode technology have significantly improved neural interfaces for both research and clinical applications. However, a significant challenge persists: scaling up these devices to achieve recording of nearly all single‐unit activity across large brain volumes. This critical review explores recent progress in neural electrode design, focusing on the challenges of achieving scalable solutions for this ambitious goal. The physical and technical constraints of both rigid and flexible probes are addressed, highlighting the limitations imposed by shank stiffness, mechanical tissue damage, and foreign body response. It is identified that the physics of inserting the electrodes into the brain tissue poses a fundamental constraint, which inherently restricts achievable electrode density. Biohybrid strategies, integrating biological and synthetic components, have shown promise, but they have yet to overcome the major challenges necessary to achieve a scalable functional interface. It is concluded that, given the current limitations of available techniques, there is a pressing need to explore fundamentally novel approaches to realize the vision of recording the electrical activity of every cortical neuron within the brain.

## Graphical abstract

Despite advancements in implantable electrode technology, challenges persist in scaling up these devices to record most of the neuronal activity across the cortex. This review explores the limitations of both rigid and flexible probes, including tissue damage and foreign body response, while also discussing emerging biohybrid strategies. It concludes by emphasizing the need for innovative approaches to achieve comprehensive neural recording.

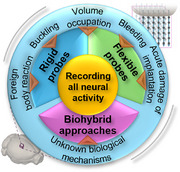

## 1 Introduction

Brain‐computer interfaces (BCIs) are systems that enable direct communication between the brain and external devices, allowing individuals to control these devices or receive feedback using neural activity. BCIs have undergone spectacular advances over the past decade;^[^ ^1^ ^]^ they are now able to restore lost physiological functions or even provide new capabilities for patients living with a variety of injuries or neurological disorders.^[^ ^2^ ^]^ In this work, we focus on reading information from the brain that can then be potentially used to perform an action at the patient's will. Recent studies showed, for example, that patients learned to use BCI to talk,^[^ ^3^, ^4^, ^5^, ^6^ ^]^ walk,^[^ ^7^, ^8^, ^9^ ^]^ or use a computer,^[^ ^10^ ^]^ which may lead to significant improvements in their quality of life. As an indicator of the rapid advancements in the field likely unlocking unprecedented possibilities, the ethical and safety implications of these technologies have also come to the forefront.^[^ ^11^, ^12^ ^]^

However, the technologies in these pioneering studies are still very much in their infancy, largely due to a significant bottleneck: the restricted amount of information that current state‐of‐the‐art devices can extract from the brain. At the moment, achievements are typically limited to a range of actions, such as pronouncing a few words, performing simple movements, or controlling gestures. These BCIs often use electroencephalography (EEG) electrodes^[^ ^8^, ^10^ ^]^ or cortical surface electrodes,^[^ ^5^, ^6^, ^9^, ^13^ ^]^ which are less invasive than penetrating implants, and rely on averaged neural signals. Although this tradeoff offers a workable solution, and even leads to consumer products for various applications,^[^ ^14^ ^]^ the complexity of what can be achieved is inevitably restricted by the low information content of the signal source.^[^ ^15^ ^]^ It is apparent that the more sophisticated human BCI accomplishments have been made possible by utilizing chronically implanted, penetrating Utah‐style probes capturing electrical signals of single neurons (e.g., the BrainGate system).^[^ ^3^, ^4^ ^]^ A number of new tools are being developed that, although not yet used in BCI systems, demonstrate significant advances and hold promise for future human BCI applications. For example, large‐scale recordings have been performed from the human brain during acute intraoperative procedures using high‐density laminar intracortical electrodes, capturing up to 100 single units per probe.^[^ ^16^, ^17^ ^]^ Others have used high‐channel‐count flexible probes and insertion shuttles to access deeply located subcortical structures in acute surgical settings.^[^ ^18^ ^]^ However, despite these cutting‐edge studies utilizing the latest techniques, these experiments have sampled only a minuscule fraction—in the order of hundreds—of the billions of neurons that constitute the human brain.

We set an ambitious goal—recording the activity of every cortical neuron—as a benchmark for testing the limits of current and emerging technologies. Although such complete coverage is beyond today's reach, using it as a target clarifies the gap between current performance and what future science and medicine may need. The exact fraction of neurons required to fully map brain function remains unknown. In some cognitive disorders, such as autism, dysfunction cannot be localized to a single region, suggesting that near‐complete, cell‐scale coverage of network activity may be necessary for deeper insight and effective interventions. Similarly, while signals from only a handful of neurons can already control basic motor actions in paralyzed patients, enabling orchestrated fine‐motor movements—or conveying nuanced expressions for communication—will likely require simultaneous recordings from several orders of magnitude more neurons. Recognizing these scientific and translational drivers, large‐scale initiatives are developing complementary technologies for uncovering structure and function on a near‐whole‐brain level.^[^ ^15^, ^19^, ^20^ ^]^ What's more, using dense electrode arrays to oversample neural tissue—where the same neuron is detected by multiple electrodes—offers key advantages such as more accurate cell‐type identification, precise 3D localization of neurons,^[^ ^21^, ^22^, ^23^, ^24^ ^]^ and improved ability to track individual neurons over long timescales.^[^ ^25^ ^]^

In this article, we will review the recent technical advancements in intracortical recording, concentrating on how far we can step toward scaling up the number of recording sites to cover most—if not all—of the neurons in a tissue volume. We will discuss the physical limitations of the different approaches, focusing on the mechanical constraints of the machine‐tissue interface and the foreign body response (FBR).

## 2 Requirements of Scalable Neuronal Recordings

The physical principles of scalable neuronal recordings are well‐established and have been summarized by Marblestone 2013.^[^ ^26^ ^]^ Although our ultimate goal is to aid human subjects, at this point, let us only consider the mouse brain, one of the most widely studied mammalian model brains. The thickness of the mouse cortex is ≈1200 µm,^[^ ^27^ ^]^ so sampling all neuronal activity within a cubic millimeter of cortical tissue would be a practical milestone toward achieving our goal. To do so, we would need to record roughly 92 000 neurons in this volume.^[^ ^28^ ^]^ The sampling rate of neural signal acquisition should be at least 1 kHz, as the lower limit required for detecting spikes. However, in this review, we do not address issues related to data sampling, transmission, and bandwidth, which have been reviewed elsewhere.^[^ ^26^, ^29^ ^]^ Additionally, in the context of this review, we do not consider subcellular or subthreshold signals.

We consider two scenarios regarding the number of electrodes necessary to sample a macroscopic brain volume. In the tolerant scenario, the activity of ≈100 individual cells may be recorded per electrode, serving as a rough estimate for the upper theoretical limit of spike sorting.^[^ ^26^ ^]^ This is supported by reports showing units recorded over 100 µm apart from the electrodes,^[^ ^30^, ^31^ ^]^ a distance well beyond the 64 µm necessary sensing distance estimated from the average cell density and spherical detection volume around the electrodes (**Table** **1**). In contrast, reliably sensing spikes from individual neurons may require shorter distances. For instance, Letner and colleagues found that neurons are distinguishable only within a range of up to 30 µm.^[^ ^32^ ^]^ Consistent with this, single‐unit yields in recent studies indicate that the number of units resolved per electrode is typically limited to about three (**Table** **2**). In line with these reports, we define the strict scenario with an electrode yield of three and a sensing distance of 20 µm.^[^ ^33^ ^]^ Table 1 summarizes the electrode yield (cells/electrode), the number of electrodes needed to capture all neurons within a cubic millimeter, and the theoretical sensing distance of the electrodes for these two scenarios. For a more detailed discussion of the limitations and opportunities related to spike sorting and electrode arrangement strategies, we refer the reader to prior comprehensive works addressing these critical aspects of high‐density recordings.^[^ ^26^, ^30^, ^33^, ^34^, ^35^ ^]^

**Table 1:** Assumption scenarios. Based on electrode yield, the number of electrodes is calculated assuming an average cell density of 92 000 cells per mm^3^. The sensing distance is calculated by considering spherical sensing volumes around the electrodes. Shank diameters are calculated assuming a 1% volume displacement of cylindrical shanks with an average length of 1 mm. For multielectrode shanks, electrodes are distributed along the 1 mm shank length with a pitch corresponding to the calculated sensing distance. The distance between the shanks should be approximately double the sensing distance.

	Unit	Tolerant scenario	Strict scenario
Reference		[26]	[33]
Cells/electrode	#	100	3
Electrodes needed/mm^3^	#	920	30 667
Sensing distance	µm	64	20
Single electrode shank max diameter	µm	3.7	0.6
Electrodes/shank	#	16	50
Multielectrode shanks needed/mm^3^	#	59	608
Multielectrode shank max diameter	µm	14.7	4.6

**Table 2:** The acute butcher number was estimated for intracortical probe approaches as described in recent studies. It is calculated as the ratio of neurons destroyed by the intervention to the number of single units recorded simultaneously. The number of neurons destroyed was calculated as the sum of the volume affected by the probe and the insertion technique, multiplied by the average cortical neuron density. For single units reported * indicates that an example or maximum value is provided, and ** indicates that a mean value across sessions is specified. In some cases, the number of units was inferred from the average unit yield statistics provided. When multiple versions of a probe were described in a study, we analyzed the version yielding the smallest butcher number. Only mammalian studies demonstrating at least three single units and studies with the probes inserted to a cortical depth of at least 400 µm were included.

Category	References	Single units reported	No. recording sites	Units / recording site	Total insertion cross‐section [µm^2^]	Kill zone volume [µm^3^]	Neurons destroyed	Butcher number	Single unit extraction remarks
Self‐supporting rigid probes									
Human clinical	[4]	80 **	96	0.83	614400	9.2E+08	23040	288.00	*manual*
	[3]	113 *	128	0.88	819200	1.2E+09	30720	271.86	*custom*
	[36]	53 **	96	0.55	614400	6.1E+08	15360	289.81	*custom*
	[37]	32 *	25	1.28	160000	2.4E+08	6000	187.50	*Plexon Offline Sorter*
	[16]	202 *	384	0.53	7000	7.0E+07	1750	8.66	*Kilosort 3.0*
	[17]	54 **	384	0.14	1400	1.4E+07	350	6.48	*Kilosort 1.0*
Conventional	[38]	260 **	1024	0.25	35200	6.7E+07	6153	23.67	*Kilosort 2.5*
	[24]	37 **	256	0.14	5000	7.7E+06	707	19.10	*Kilosort 2.0*
	[39]	200 *	384	0.52	1680	1.0E+07	927	4.64	*Kilosort 2.5*
	[40]	50 **	54	0.93	3180	3.2E+06	293	5.85	*custom*
Carbon fiber	[41]	57 **	34	1.68	1883	2.3E+06	208	3.65	*Plexon Offline Sorter*
	[42]	17 **	12	1.42	665	6.1E+05	56	3.28	*Plexon Offline Sorter*
	[43]	37 **	32	1.16	1061	6.4E+05	59	1.58	*MountainSort*
	[44]	22 *	20	1.10	769	6.9E+05	64	2.90	*Plexon Offline Sorter*
	[32]	23 *	16	1.44	886	1.1E+06	98	4.25	*Plexon Offline Sorter*
Silicon etch	[45]	259 *	256	1.01	8800	5.7E+07	5262	20.32	*Kilosort 2.0*
Other	[46]	221 *	251	0.88	56943	5.7E+07	5239	23.70	*MountainSort*
	[47]	791 *	1300	0.61	330642	2.8E+08	25856	32.69	*WaveClus*
Flexible probes with implantation support									
Bulk insertion	[48]	14 *	16	0.88	331663	3.3E+08	30513	2179.50	*WaveClus*
	[49]	31 *	16	1.94	237463	4.7E+08	43693	1409.45	*WaveClus 2.0*
	[50]	13 *	16	0.81	17663	1.8E+07	1625	125.00	*Clampfit*
	[51]	38 **	128	0.30	5024	1.0E+07	924	24.07	*Plexon Offline Sorter*
	[52]	25 **	16	1.56	14400	1.4E+07	1325	52.99	*MountainSort*
Shuttle device	[53]	40 **	32	1.25	1094	8.8E+05	81	2.01	*Kilosort*
	[22]	1355 *	1024	1.32	34280	3.4E+07	3154	2.33	*MountainSort*
	[54]	20 *	60	0.33	220	3.3E+05	30	1.52	*KlustaKwik2*
	[55]	1401 **	3072	0.46	52623	1.3E+08	11619	8.29	*custom*
Magnetic	[56]	5 *	6	0.83	2944	1.1E+07	1002	200.41	*OSort*
					Ideal butcher number <			0.05	

**Figure 1:**
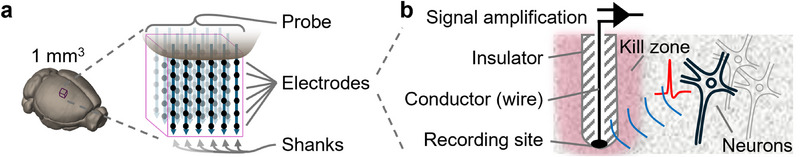
Schematics of electrode placement in the cortex. a) Probes are made of one or more shanks, which are the protrusions that actually penetrate the tissue. They are sometimes also referred to as threads. Shanks support one or more electrodes along their length, which serve as the recording sites for the electrical signals of nearby cells. b) Main components of an electrode within the tissue. Perturbation of the electromagnetic field by the cell's action potential is sensed by the recording sites up to ≈140 µm distance.^[^ ^30^ ^]^ “Kill zone” is marked with a purple area around the electrode.

Wires from each electrode must reach the surface of the brain via the shanks, displacing some volume (**Figure** **1**). Here, we assume a total volume displacement of less than 1% from the shanks.^[^ ^26^ ^]^ Based on this assumption, we can calculate a submicrometer diameter for single‐electrode shanks in the strict scenario (Table 1). To optimize volume displacement, shanks can be designed to have multiple electrodes, thereby reducing the number of shanks required. As the electrode spacing on the shanks should ideally be comparable to the sensing distance, the maximum permitted shank diameter is ≈5 µm for multielectrode shanks in the strict scenario (Table 1). These values represent approximate target diameters for the shanks, which should be maintained along the entire length of the shank and also include surrounding areas that may have been damaged during insertion.

To quantify the damage caused by an interface during the intervention, we introduce a simple dimensionless term: the butcher number, defined as the ratio of neurons destroyed to the number of single units recorded simultaneously. While it is a roughly defined measure, it provides a useful estimate of how far an approach has progressed. To achieve our objective of recording all neurons, this number must converge to zero, or more realistically, remain within just a few percent, so that most cortical activity can be studied with minimal perturbation. Building on this definition, we may further distinguish between the acute butcher number, which accounts for neurons destroyed during the initial intervention (e.g., probe implantation), and the long‐term butcher number, which in addition accounts for neurons lost over chronic timescales (e.g., due to FBR). The acute butcher number can be reasonably estimated from geometrical considerations, such as the space occupation of the probe. In contrast, chronic damage is often effectively mitigated in the reviewed state‐of‐the‐art studies using biocompatible materials and extensive miniaturization. For this reason, we focus primarily on the acute phase of tissue damage when estimating the butcher number. In the following sections, we review recent studies that have made significant progress toward scaling up cortical recordings, and in doing so, we also examine the butcher number, as summarized in Table 2.

## 3 Self‐Supporting Rigid Probes

**Figure 2:**
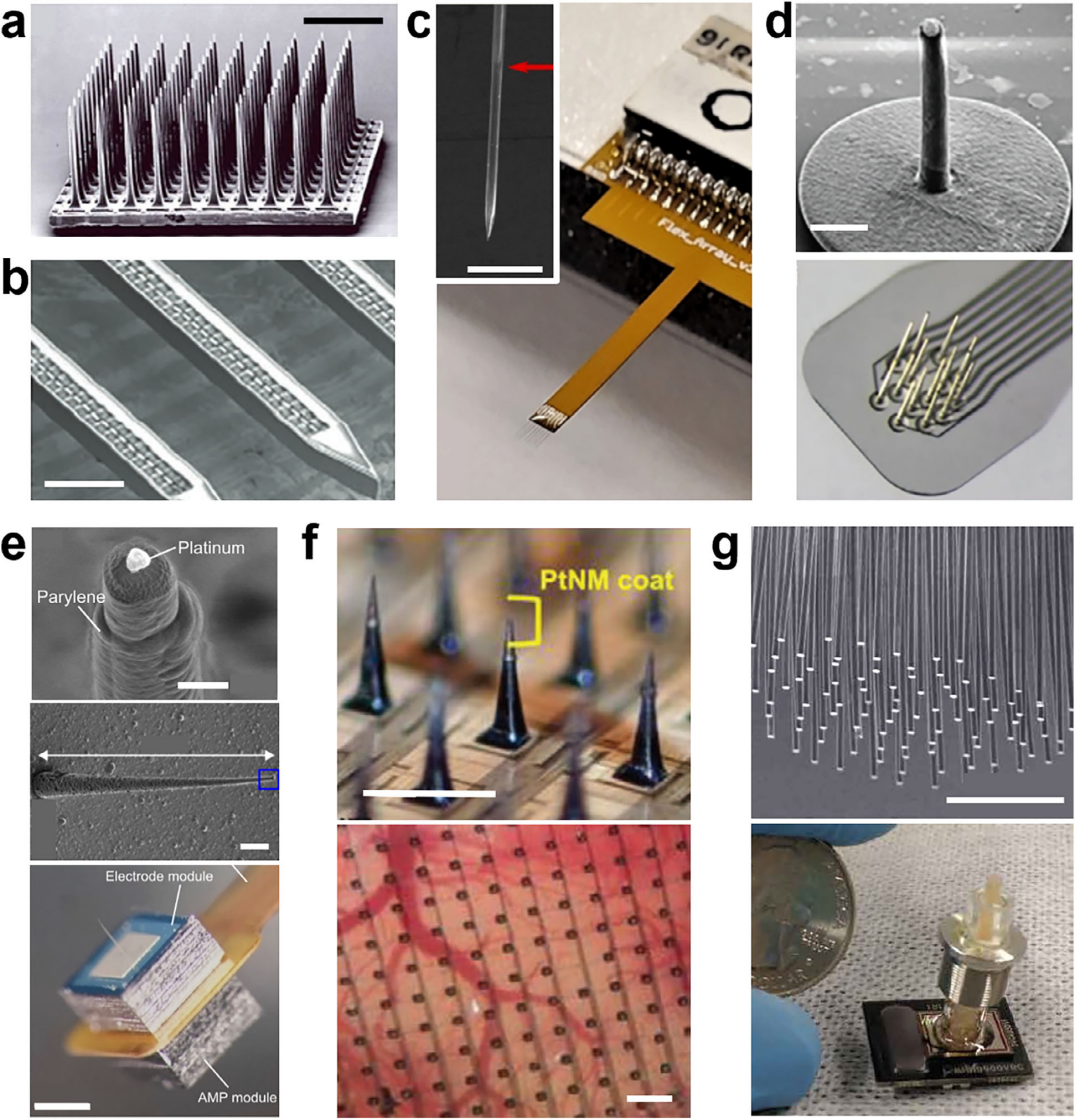
Examples of self‐supporting rigid probes. a) Scanning electron microscope (SEM) image of a Utah‐array (BrainGate). Scale bar, 1 mm. Adapted with permission.^[^ ^36^ ^]^ Copyright 2006, Springer Nature. b) Michigan‐style probe (SiNAPS). Scale bar, 150 µm. Adapted under terms of the CC‐BY license.^[^ ^38^ ^]^ Copyright 2025, The Authors, published by Wiley‐VCH. c) Carbon fiber electrode array. (inset) SEM image of one sharpened fiber end shows the transition between coated and bare carbon fiber (red arrow). Scale bar, 50 µm. Adapted with permission.^[^ ^63^ ^]^ Copyright 2021, JoVE. d) (top) 3D printed electrode. Scale bar, 20 µm. (bottom) Array formed of these. Adapted under terms of the CC‐BY license.^[^ ^64^ ^]^ Copyright 2024, The Authors, published by Wiley‐VCH. e) Three‐micrometer‐tipped silicon needle‐electrode device. Scale bars, 2, 20, 500 µm. Adapted with permission.^[^ ^65^ ^]^ Copyright 2021, PNAS. f) 1024‐channel silicon microneedle array on a flexible substrate. Scale bars, 500 µm. Adapted with permission.^[^ ^66^ ^]^ Copyright 2022, Wiley‐VCH. g) Large‐scale microwire array. Scale bar, 500 µm. Adapted under terms of the CC‐BY license.^[^ ^46^ ^]^ Copyright 2020, The Authors, published by AAAS.

The first high‐channel‐count, high‐density electrode solutions developed were rigid, silicon‐based electrode arrays, fabricated using different manufacturing techniques, namely the Utah arrays (**Figure** **2**a)^[^ ^36^, ^57^ ^]^ and the Michigan‐style probes (Figure 2b).^[^ ^38^, ^39^, ^40^ ^]^ For instance, Neuropixels silicon probes are among the most advanced Michigan‐style probes and are widely used by the neuroscience community in both human subjects and various animal models.^[^ ^16^, ^39^ ^]^ These devices feature thousands of densely packed recording sites, enabling the simultaneous recording of hundreds of well‐isolated neurons with a high signal‐to‐noise ratio in acute and chronic settings. Both Utah and Michigan probes are sufficiently stiff to be inserted into the tissue without additional mechanical support, and some have also been used in the aforementioned human investigations.^[^ ^3^, ^4^, ^16^ ^]^ However, numerous studies have reported that these rigid probes elicit a marked FBR and glial scar in the surrounding tissue,^[^ ^58^, ^59^ ^]^ which leads to a gradual deterioration in signal quality over time.^[^ ^60^, ^61^ ^]^ During this process, most neurons and other tissue elements are lost in the region up to 100 µm around the shanks, often referred to as the “kill zone” (Figure 1b).^[^ ^62^ ^]^ As shown in Table 2, the acute butcher number of these interventions ranges from 4 to 300, which is further worsened by long‐term tissue degradation effects. The two effects combined limit the increase in shank density,^[^ ^37^ ^]^ indicating that rigid probes—typically spaced hundreds of microns apart—cannot target large tissue volumes without the cumulative kill zone impacting the entire area. Such widespread disruption compromises physiological network activity, limiting application for comprehensive activity recording.

Ensuring the long‐term functionality and chronic reliability of implanted neural interfaces is a critical aspect that needs to be taken into consideration. The major factors to address include minimizing insertion‐related damage to the blood‐brain barrier, reducing micromotions, and mitigating the foreign body response. While many studies actually follow cellular activity over long timescales,^[^ ^48^, ^52^, ^61^ ^]^ FBR and glial scarring are typically assessed using histological markers of neuron loss and of increased number of astrocytes and microglia.^[^ ^1^, ^67^, ^68^, ^69^, ^70^, ^71^ ^]^ Several recent reviews have discussed in detail the most important strategies and aspects to improve long‐term performance.^[^ ^1^, ^67^, ^72^ ^]^ These include refining implantation procedures—such as optimizing insertion speed^[^ ^73^ ^]^ and force,^[^ ^74^ ^]^ employing neurovascular mapping,^[^ ^75^ ^]^ and improving probe fixation methods—as well as advancing device design through adjustments to probe size, layout, tip geometry, and mechanical properties.^[^ ^76^, ^77^ ^]^ The selection of materials for the substrate and recording sites also plays a significant role.^[^ ^78^, ^79^ ^]^ Furthermore, enhancing biosafety and biostability through the use of biocompatible materials, bioactive surface coatings, or localized drug delivery are also key areas of focus.^[^ ^72^, ^80^ ^]^

The most effective approach to mitigating FBR is to reduce the cross‐sectional area of probe shanks, acting through complementary mechanisms. Probes with smaller surgical footprints are less likely to damage capillaries during insertion.^[^ ^81^ ^]^ Thin electrodes, even when composed of stiff materials, remain flexible, allowing them to better accommodate tissue motion and therefore reduce the FBR.^[^ ^71^, ^82^ ^]^ Furthermore, they reduce the adsorption of biomolecules that potentially cause gliosis and have less impact on the normal perfusion of the neural tissue.^[^ ^67^ ^]^ Therefore, in the following section, we discuss recent technological advancements that have led to the development of the thinnest self‐supporting probes to date.

Several studies have explored the use of electrodes made from carbon fiber (Figure 2c).^[^ ^83^ ^]^ These studies typically utilize fibers with diameters ranging from 4 to 8 µm,^[^ ^84^ ^]^ which are usually insulated with Parylene‐C. Various methods have been developed to cut^[^ ^41^ ^]^ and shape^[^ ^42^ ^]^ the electrode tips. While the method is easy to implement for a few electrodes,^[^ ^63^ ^]^ it has also been scaled up to 64 electrodes.^[^ ^43^, ^44^ ^]^ These probes offer a good compromise in terms of stiffness: they are thinner and more flexible than silicon probes, which contributes to their minimal FBR, yet stiff enough to be inserted into the tissue to depths of several millimeters.^[^ ^81^ ^]^ Multiple studies have evaluated the glial scar caused by these single‐electrode probes, demonstrating that they perform significantly better than rigid silicon probes tens of microns wide^[^ ^81^, ^82^ ^]^ and provide stable recordings over chronic timescales.^[^ ^82^ ^]^

Thin probes can be constructed by 3D printing the outer insulating sheath of the electrode, and then filling this sheath with an aluminum conducting core in a subsequent step (Figure 2d).^[^ ^64^ ^]^ This method provides great flexibility in probe design while avoiding the costs associated with silicon microfabrication. Abu Shihada and colleagues reached a diameter of 12 µm at the electrode tip and created tapered electrodes up to 500 µm long. The construct was inserted into the cortex of mice and showed adequate signal quality in vivo. Thorough deflection tests and modeling showed that this technology approached the thinnest construct that could withstand the insertion process. Using silicon etching technology, Kita and colleagues achieved a significant milestone by fabricating single‐electrode, single‐shank neural probes that are 400 µm long with a tip diameter of 3 µm (Figure 2e).^[^ ^65^ ^]^ To ensure adequate mechanical strength despite using strong silicon material, they designed the probe with a conical shape that widens to a diameter of 25 µm at the base. They argued that the thinness of the probe minimizes tissue damage and reported minimal cellular alterations in a two‐week experiment. Additionally, they successfully integrated an amplifier onto the base of the probe to correct for the electrical properties of the tiny structure, resulting in adequate signal collection properties in vivo.

Increasing the number of recording sites with these thin, self‐supporting probes is essential to achieving our goal. To this end, a Utah‐style 1024‐channel microneedle array has been developed, composed of a flexible polymer backplane and microneedles fabricated by silicon etching (Figure 2f).^[^ ^66^ ^]^ The functionality of the array was verified in vivo. The microneedles were 300 µm long, sharp at the tip, and had a tapered shape, reaching a diameter of ≈50 µm at their base to improve the mechanical stability during insertion. Similarly, another group demonstrated an ingenious design suitable for clinical translation by developing a microwire array consisting of 251 pieces of 15 µm diameter PtIr microwires with ≈100 µm separation. These metal wires were interfaced in a single step with a modified CMOS chip eventually yielding 221 putative single units (Figure 2g).^[^ ^46^ ^]^ This technology was later scaled up to an array of 30 000 electrodes with a pitch of 60 µm.^[^ ^47^ ^]^

**Figure 3:**
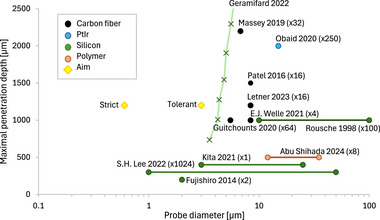
An overview of the properties of reported single‐electrode self‐supporting probes. Horizontal lines represent the tip and base diameters of tapered designs. The number of shanks per probe is denoted by parentheses. Colors indicate the material of the load‐bearing structure: (black) carbon fiber, (blue) PtIr, (green) silicon, (brown) polymer. Targeted single‐electrode shank diameters calculated from theoretical approaches are marked in yellow. The diagonal curve represents the maximum possible length of silicon carbide probes that can be inserted into the brain, as derived from Figure 11 of Geramifard 2022.^[^ ^85^ ^]^

The common characteristic of these studies utilizing single‐electrode shanks is that even the thinnest shanks do not achieve the target diameter of 3.7 µm throughout their length, as required in the “tolerant” scenario, and fall far short of the 0.6 µm diameter specified in the “strict” assumption (Table 1 and **Figure** **3**). Importantly, there is a material‐based physical limitation rather than a technical one: further thinning of the shanks would cause them to buckle during the insertion process. Numerous investigations have measured and modeled the buckling and related maximal force that the probe can withstand without buckling during implantation.^[^ ^64^, ^81^, ^85^, ^86^ ^]^ These studies point out that the minimum diameter for a probe to be inserted into the cortex without external support is ≈5 µm, even though buckling can be mitigated during the implantation process using microfluidic actuators,^[^ ^87^ ^]^ insertion guides,^[^ ^88^, ^89^ ^]^ or ultrasonic vibrations.^[^ ^90^ ^]^ Further reducing the shank diameter from the 5 µm range down to the 0.6–3.7 µm range (as required by our theoretical calculations), while seemingly straightforward, presents a significant challenge. The bending stiffness of the shank scales with the fourth power of its diameter, so this reduction in size would drastically lower the tolerated insertion force. Experimental insertion studies have shown that this would be clearly insufficient to successfully penetrate brain tissue.

Arranging electrodes to form multielectrode shanks allows the shanks to reach diameters of several micrometers, as determined by our theoretical calculations on maximum volume occupation (Table 1). This approach could address the issue of insufficient stiffness for insertion while maintaining high electrode density and minimal tissue compression. For example, laminar‐fabricated probes designed to withstand implantation without external support recently achieved shanks with a cross‐sectional area of 5 × 10 µm^2^ and a spacing of only 66 µm between shanks (arranged in a row), resulting in 256 electrodes per probe.^[^ ^45^ ^]^ In in vivo experiments, 259 single units were recorded simultaneously; however, post hoc histological staining for FBR markers was not performed in this study. Eight electrodes were formed along the first 200 µm of the probe shanks, while the base regions of the shanks were widened to 25–40 µm to provide adequate support for implantation into the tissue. If we consider only the narrower shank region containing the electrodes, this design approaches the 1% volume occupation goal. However, the tapered probe design indicates that the mechanical limitations of the technology restrict any substantial further increase in penetration depth. Moreover, by choosing such a relatively thick, rigid multielectrode solution, we take a step back in our efforts to develop thinner shanks to minimize the FBR. Let us, therefore, investigate whether the FBR allows for scaling up rigid multielectrode approaches to enable full sampling of larger tissue volumes, as previously envisioned.^[^ ^33^ ^]^

**Figure 4:**
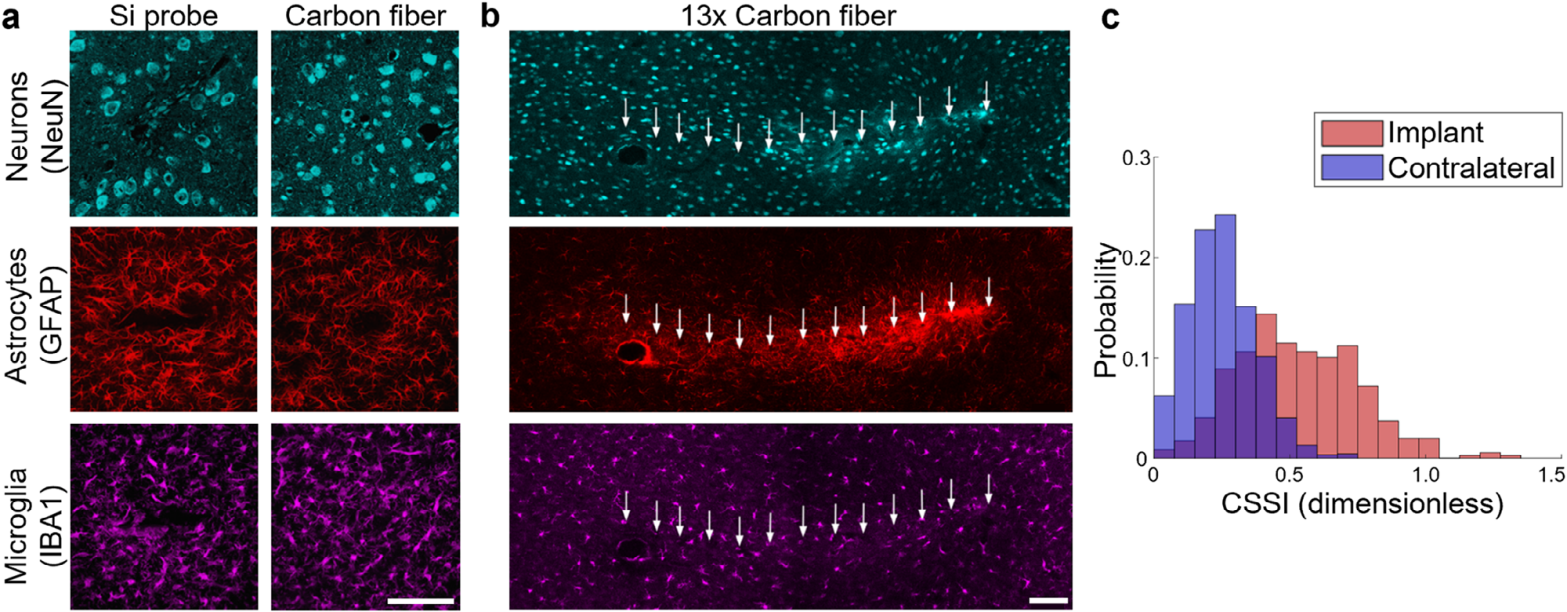
Tissue consequences caused by the implantation of micrometer‐scale devices. a) Histological comparison of tissue reactions to chronically implanted carbon fiber electrodes and conventional silicon probes in the motor cortex following a two‐week implantation. Adapted with permission.^[^ ^81^ ^]^ Copyright 2012, Springer Nature. b) Confocal histology image from layer V of the rat motor cortex after explantation of 13 carbon fiber electrodes following 12 weeks of implantation. Glial responses aided in the localization of electrode tracts, marked by white arrows. (a,b) Cyan: NeuN (neurons); red: GFAP (astrocytes); magenta: Iba1 (microglia). Scale bars, 100 µm. c) Histograms showing the cell shape strain index (CSSI) calculated for the neurons located within 50 µm from the recording site tips and in the contralateral tissue without an implant. CSSIs around carbon fibers were significantly larger, indicating that the neurons were stretched. (b,c) Adapted under terms of the CC‐BY license.^[^ ^32^ ^]^ Copyright 2023, The Authors, published by IOP Publishing.

Studies on the FBR to thin probes (5–10 µm in width) typically compare the effects of a single thin shank on the tissue with a much wider (>50 µm) shank of a rigid silicon probe (**Figure** **4**a).^[^ ^46^, ^70^, ^71^, ^74^, ^81^, ^82^ ^]^ Alternatively, they demonstrate that a single shank does not elicit a tissue response distinguishable from intact tissue.^[^ ^41^, ^65^ ^]^ The use of 32 shanks (100 µm long, 10 µm wide) placed 400 µm apart caused inflammation comparable only to that caused by a craniotomy with durotomy alone.^[^ ^66^ ^]^ While these studies do not show marked FBR to a small number of thin shanks, the question remains whether scaling up the number of shanks to thousands or more could introduce challenges associated with FBR. The mechanisms underlying FBR and glial scar formation involve multiple pathways, which may exhibit varying sensitivity to the diameter and density of probe shanks. Consequently, it has been questioned whether merely reducing probe shank diameters below 10 µm is sufficient to achieve a scalable solution for large‐scale electrode arrays.^[^ ^67^, ^69^, ^91^ ^]^

A recent study analyzed in detail the tissue alterations following the implantation of sixteen Parylene C‐coated carbon fiber intracortical electrodes having a diameter of 7 µm and a pitch of 80 µm (Figure 4b).^[^ ^32^ ^]^ It is important to highlight that the localization of the electrodes after explantation was largely based on the FBR markers that are overlooked by others. Since already a few shanks positioned in a row can cause a measurable FBR, the insertion of thousands of shanks in 3D is expected to severely affect the tissue due to inflammatory pathways, which are likely to reach their activation threshold.^[^ ^79^, ^92^, ^93^ ^]^ Therefore, it is highly unlikely that recording the activity of most neurons can be achieved by inserting thousands of rigid shanks with several micrometer diameters.

Besides FBR characterized by standard markers, implantation can cause a shift toward inhibitory activity,^[^ ^94^ ^]^ hypoxia and progressive neurite degeneration,^[^ ^95^ ^]^ myelin injury and loss of oligodendrocytes,^[^ ^96^ ^]^ elevated extracellular ATP level^[^ ^97^ ^]^ and mechanical distortion of nearby neurons (Figure 4c).^[^ ^32^ ^]^ RNA sequencing of the tissue at implant sites revealed differential expression of over 100 genes, indicating that FBR induces complex cellular changes.^[^ ^98^ ^]^ Given these complex reactions of many different types and the fact that most existing studies address the effects of larger devices (>50 µm), extrapolating these findings to scenarios involving numerous small fibers is challenging.^[^ ^99^ ^]^ Further studies are needed to quantify these mechanisms within the context of our specific scenario.

In summary, a major limitation of self‐supporting probes is their need for sufficient shank thickness and stiffness to ensure successful tissue penetration. However, these properties are also the primary triggers of FBR, limiting the scalability of such approaches for detecting most of the neuronal activity within a tissue volume. Further reduction in electrode shank thickness compromises their ability to penetrate tissue independently. Therefore, in the following section, we explore strategies involving mechanically supported implantation of thin flexible probes.

## 4 Flexible Probes with Implantation Support

Minimizing the mechanical mismatch between probes and brain tissue significantly reduces the chronic aspects of the FBR. In addition to geometric factors, intrinsic material properties such as Young's modulus have been shown to significantly influence the FBR, with softer materials generally eliciting a milder response.^[^ ^77^ ^]^ The benefits of such probe designs have been reviewed in several recent publications,^[^ ^67^, ^76^ ^]^ showing their promise in creating large‐scale and stable interfaces. These advanced technologies combine miniaturization and flexibility of the shanks (often referred to as threads or fibers) to record single‐unit activities over chronic timescales, while their insufficient rigidity for insertion is addressed using specialized implantation methods. Common approaches for implanting these probes that were fabricated outside the tissue include the use of glass capillaries, stiffeners, or shuttle devices.^[^ ^100^, ^101^ ^]^

**Figure 5:**
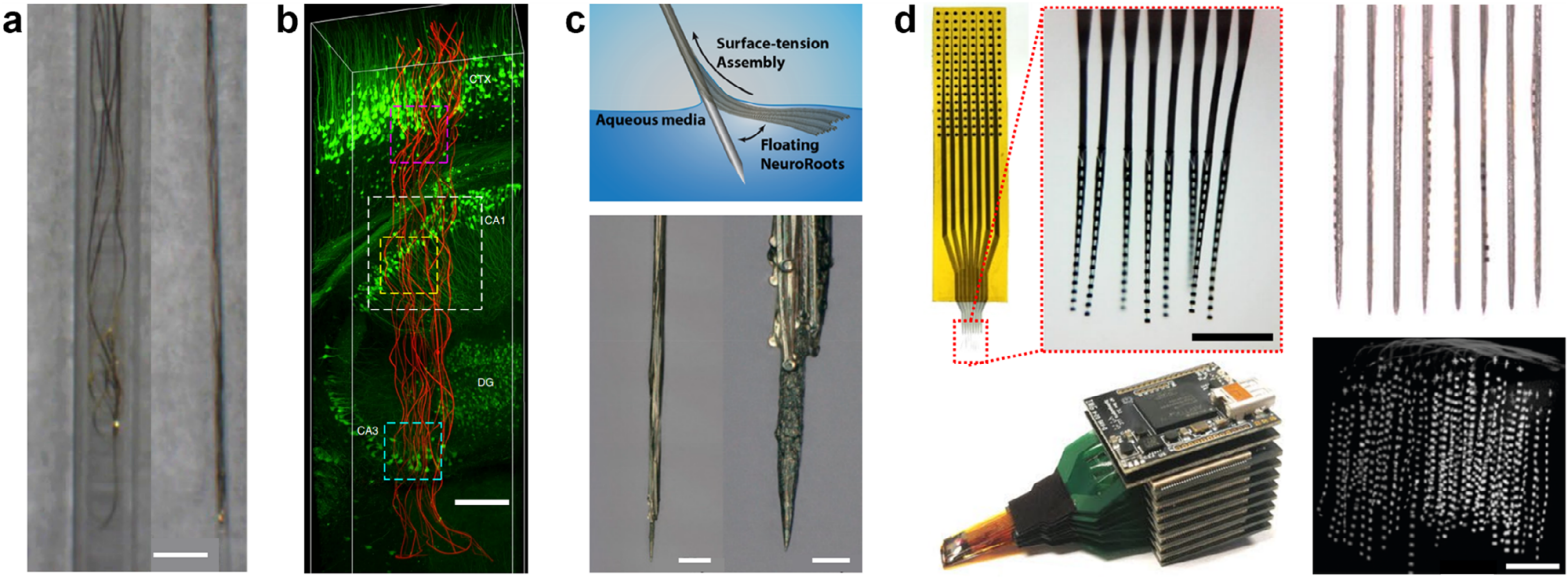
Examples of flexible probes with implantation support. a) Mesh electronics prepared for injection in 400 µm/650 µm and 100 µm/170 µm ID/OD glass capillary needles. Scale bar, 500 µm. Adapted with permission.^[^ ^103^ ^]^ Copyright 2019, American Chemical Society. b) Neuron‐sized electronics mapped 6 weeks after implantation with a capillary. Scale bar, 200 µm. Adapted with permission.^[^ ^49^ ^]^ Copyright 2019, Springer Nature. c) NeuroRoots probe with ultrathin electrodes glued to a 100 µm diameter microwire using PEG. Scale bars, 100 µm, and 25 µm. Adapted under terms of the CC‐BY license.^[^ ^104^ ^]^ Copyright 2024, The Authors, published by AIP Publishing. d) (top) 8‐shank, 128‐channel flexible multielectrode module. The right panel shows individual shanks attached to tungsten microwires for implantation. (bottom) The 1024‐channel system is composed of eight modules. (bottom right) Micro‐CT scan showing the volumetric distribution of the electrodes in the mouse visual cortex. Scale bars, 500 µm. Adapted with permission.^[^ ^22^ ^]^ Copyright 2022, Springer Nature.

**Figure 6:**
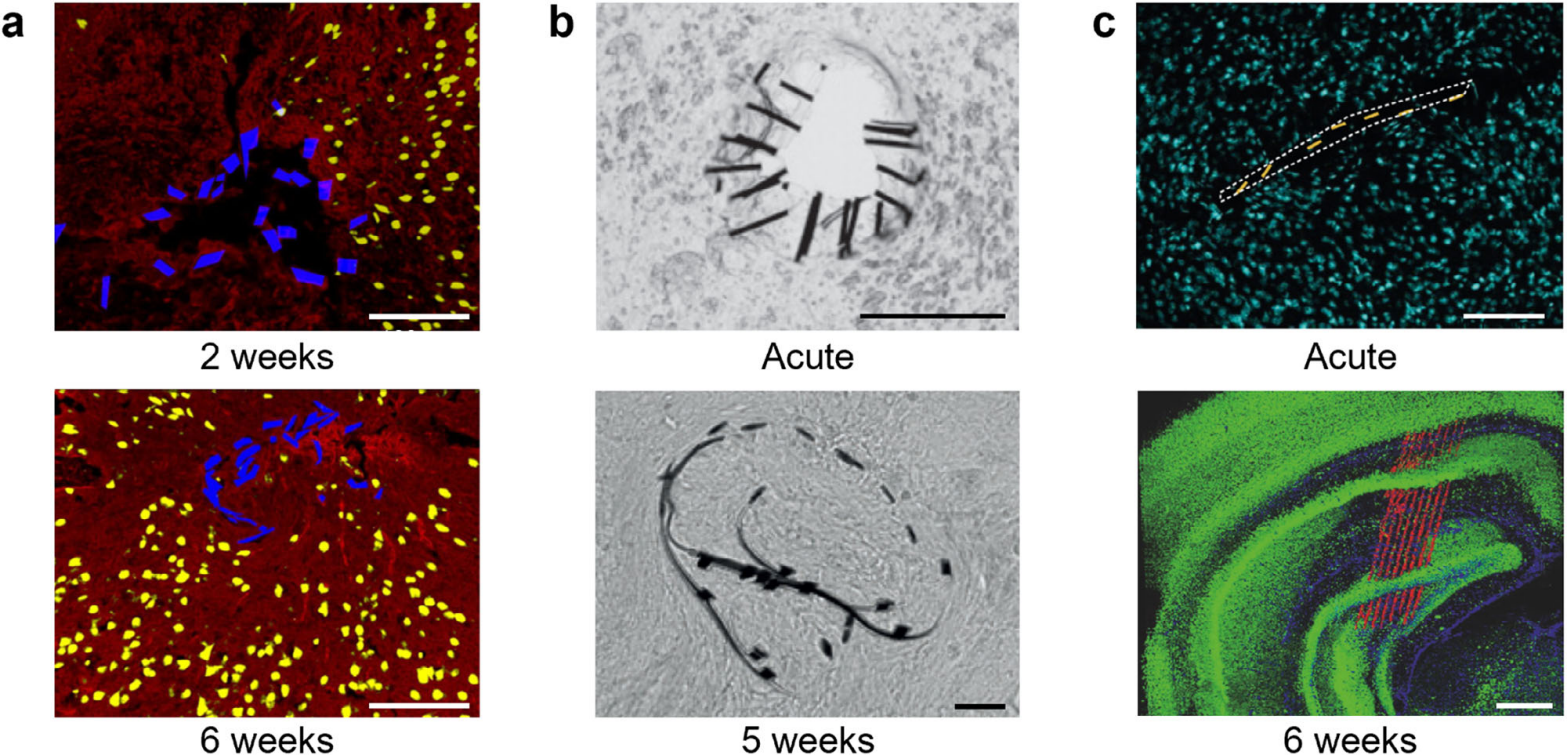
Acute damage caused by implantation of mesh probe structures. a) Immunohistochemistry images at 2 weeks (top, hippocampus), and 6 weeks (bottom, cortex) after injection of the probe. Red, yellow, and blue correspond to neurofilaments, NeuN, and probe, respectively. Scale bars, 100 µm. The acute damage caused by the 650 µm outer diameter needle used for injection is partially healed after 2 weeks. Adapted with permission.^[^ ^48^ ^]^ Copyright 2016, Springer Nature. b) Brightfield images of the implanted probe/tissue interface cross‐section where the probe was stiffened by rapid freezing in liquid nitrogen after being removed from a buffer solution. The process results in a 100–200 µm diameter cylinder. Dark regions in the image represent the components of the probe. Acute slice (top; scale bar, 100 µm) and a slice five weeks after implantation (bottom; scale bar, 20 µm). Adapted with permission.^[^ ^50^ ^]^ Copyright 2015, Springer Nature. c) Mesh probe implanted with a planar polymer shuttle. Image of a horizontal brain slice showing the acute tissue damage introduced by the shuttle (top). Yellow and cyan represent cross‐sections of the probe and DAPI staining, respectively. The tissue damage region is highlighted by white dashed lines. (bottom) 3D‐reconstructed confocal fluorescence images 6 weeks after implantation, illustrating cuts across multiple brain regions caused by probe implantation. Scale bars, 400 µm. Adapted with permission.^[^ ^52^ ^]^ Copyright 2023, Springer Nature.

Several research groups have designed flexible neural probes with widths comparable to or smaller than the average cell. These devices form reliable and glial scar‐free neural‐probe interfaces due to the probe‐tissue interfacial forces being within the range of cellular forces. These probes have demonstrated year‐long single‐unit recordings,^[^ ^48^, ^52^ ^]^ stabilized in place by cellular processes weaving around the fibers. However, implantation of these fine, fibrous structures poses a challenge, as individual fibers are prone to bending, and many of these fibers need to be spread within the tissue. One approach is to load the probe into a relatively large glass capillary tube, insert the entire device into the tissue, and then push out the fibers while retracting the tube (**Figure** **5**a,b).^[^ ^49^, ^102^, ^103^ ^]^ A study carefully examining the effect of various needle sizes demonstrated successful insertion with needles as small as 100 µm/170 µm (ID/OD), resulting in reduced tissue damage.^[^ ^103^ ^]^ This method leaves an over 100 µm diameter void in the tissue, where the tube used to be, and places the electrodes into this wound that needs to heal (**Figure** **6**a).^[^ ^48^ ^]^ Consequently, one must calculate the acute butcher number not based on the dimensions of the probe, but based on the size of the insertion guide, frequently resulting in a high butcher number reaching up to 1000 (Table 2), contradicting the original aim of minimizing the invasiveness of the probe.

An alternative approach that yields similar results involves freezing the electrodes,^[^ ^50^ ^]^ applying a bioresorbable polymer shuttle that dissolves after insertion,^[^ ^105^ ^]^ or gluing the electrodes together using polyethylene glycol (PEG) into a rod‐like bundle.^[^ ^51^ ^]^ As ice, PEG, and other stiffeners^[^ ^106^ ^]^ have lower tensile strength compared to carbon fibers or metals, these structures need to be relatively thick to withstand implantation, practically exceeding 100 µm in diameter. As a result, following the dissolution of the temporary adhesive material or melting of the ice, again a large tissue void remains where the electrodes are distributed (Figure 6b).^[^ ^50^ ^]^ Others adhered the microfiber mesh to a plate‐like polymer shuttle to insert it in a spread‐out form.^[^ ^52^ ^]^ For this reason, the insertion device had a wide cross‐section (25 × 500 µm^2^), cutting across multiple brain regions and leaving again extensive network damage around the fine electrodes (Figure 6c).

Naturally, these acute wounds heal, and the signatures of the acute inflammatory response to the stab wound typically resolve almost entirely to the pre‐injury state within 16 weeks,^[^ ^107^ ^]^ resulting in normal neuron count and minimal FBR over chronic timescales.^[^ ^48^, ^49^, ^52^ ^]^ Our goal, however, is to scale up the technology to enable mapping of the entire tissue, a challenge that becomes unfeasible if the probe implantation inevitably damages the volume spanned by the electrodes. To reduce the amount of this damage, insertion supports should be as thin as possible, and consequently, made of materials that are the most resilient to bending and braking. To do so, each flexible electrode shank is temporarily attached to a rigid shuttle device, often made of a thin metal wire; during implantation, the shank and the shuttle are pushed into the tissue together, after which the shuttle is withdrawn, leaving the electrodes in place.

Here, we present recently published approaches utilizing flexible probes combined with the thinnest shuttle devices. A probe having 32 electrodes per shank was fabricated out of a flexible polyimide thin film, achieving a small cross‐section (10 × 78 µm^2^) and demonstrating tens of single units per shank in vivo.^[^ ^53^ ^]^ Implantation used a 20 µm diameter glass fiber as a shuttle device, with biodissolvable PEG as a temporary adhesive between the probe and the shuttle. Another group fabricated an ultrathin, flexible, bidirectional probe (recording and stimulating) having 32 electrodes across two shanks. Each shank had a cross‐section of 2.5 × 100 µm^2^ and was implanted simultaneously into both hemispheres using PEG‐adhered 50 µm diameter tungsten wires. Electrodes enhanced with a PEDOT:PSS/IrO_x_ composite allowed also the delivery of stimulus pulses, which were shown to alter the animal's behavior.^[^ ^108^ ^]^

**Figure 7:**
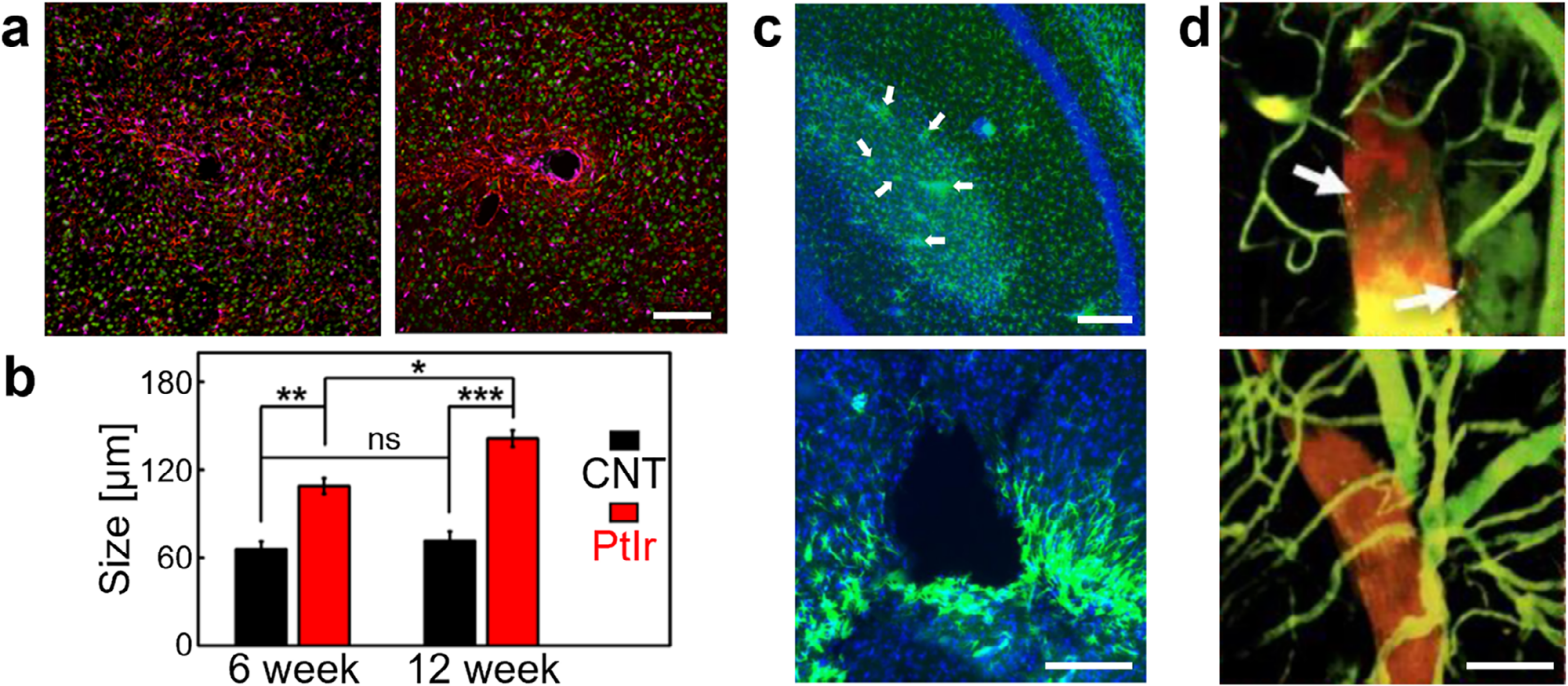
Probes implanted by shuttle wires. a) Immunofluorescence images of tissue responses 6 weeks after implantation of a CNT fiber microelectrode (20 µm diameter, left) and a PtIr wire electrode (25 µm diameter, right). Green: NeuN (neurons); red: GFAP (astrocytes); purple: Iba1 (microglia). Scale bar, 100 µm. b) Neuron kill zone diameters for CNT fiber and PtIr implants 6 and 12 weeks after implantation. The neuron kill zone for CNT fiber electrodes is comparable to the implantation footprint (≈20 µm diameter CNT fiber insulated with ≈2.5 µm thick Parylene‐C plus ≈50 µm tungsten microwire shuttle), indicating that there was no further neuronal degeneration beyond the acute tissue damage from the implantation. a,b) adapted with permission.^[^ ^109^ ^]^ Copyright 2019, American Chemical Society. c) Histology 90 days after the implantation of PEDOT:PSS‐coated NeuroRoots probe with a 100 µm diameter microwire. GFAP staining (green) shows reactive astrocytes highlighting probe tips (top, white arrows). DAPI (blue) labels cell nuclei. Tissue response along the trajectory of the implant in the cortical region (bottom). Scale bars, 100 µm. Adapted under terms of the CC‐BY license.^[^ ^104^ ^]^ Copyright 2024, The Authors, published by AIP Publishing. d) 3D reconstruction of two‐photon microscope images of the capillary network (green) surrounding the same 1 × 50 µm^2^ flexible probe shank (red) immediately (top) and 2 months (bottom) after implantation. Local bleeding was observed acutely (highlighted by arrows), which completely disappeared over time. The probe also underwent position changes, and significant vasculature remodeling occurred. Scale bar, 50 µm. Adapted with permission.^[^ ^111^ ^]^ Copyright 2017, AAAS.

To further decrease shank cross‐section, soft and MRI‐compatible neural electrodes were developed using Parylene‐C coated carbon nanotube (CNT) fibers with a diameter down to 10 µm.^[^ ^109^ ^]^ Implantation utilized a 50 µm diameter tungsten wire and poly(ethylene oxide) (PEO) as an adhesive. The study showed reduced neuron kill zones and glial markers compared to rigid PtIr wires of the same diameter. However, this reduced zone was still comparable to the implantation footprint, indicating that the neuron loss was the result of the acute tissue damage caused by the probe and the tungsten wire (**Figure** **7**a,b). Others reduced electrode diameters even further to match the size, flexibility, and distribution of axons (Figure 5c).^[^ ^104^ ^]^ A dense array of 32 such electrodes was implanted into the brains of freely moving rats using sharpened microwire shuttles, achieving long‐term stable recordings. In these cases, tissue damage was primarily dominated by the footprint of the shuttle device (Figure 7c).

The most promising report toward our aim demonstrated ultrathin electrodes in a 3D interface, providing 1024 recording sites and isolating 987 single units within 1 mm^3^ of the cortex (Figure 5d).^[^ ^22^ ^]^ The configuration includes 16 electrodes on each shank, forming eight‐shank probes. Eight such probes were inserted into the animal's brain in close proximity to each other with an innovative process. Each shank, with a cross‐section of 1 × 25 µm^2^, was temporarily attached to a 25 µm diameter tungsten wire with PEG. This technique achieved an electrode density comparable to that of our tolerant scenario. However, even assuming that up to 90% of the cells are silent or show sparse firing,^[^ ^110^ ^]^ the system captured only 5–10% of all active neurons within the covered volume. Nevertheless, these neural recordings represent an exceptionally rich dataset. Largely accounted for the shuttle wires, at least 4% of the tissue volume was destroyed during the implantation, leading to an acute butcher number in the order of two (Table 2). Achieving electrode densities close to our strict assumption to sample all neurons would require further increase in shank density by several factors, leading to a significant percentage of the tissue being destroyed during the insertion process and causing a critical reorganization of the targeted tissue. Earlier work by the same group^[^ ^111^ ^]^ used even smaller probes (1 × 10 µm^2^) with carbon fiber shuttles, to reduce the total implantation cross‐section to 10 × 10 µm^2^. In this study, the authors used two‐photon microscopy to visualize the healing of vascular damage post‐implantation, observing a recovery of the vasculature within 4 weeks (Figure 7d).

As demonstrated, the cross‐section of flexible electrode shanks has been scaled down significantly, meeting the theoretical threshold for occupied tissue volume. Furthermore, these electrodes have proven adequate sensing functionality and biocompatibility over chronic timescales. However, the insertion shuttles remain constrained by the stiffness requirements of the implantation procedure and therefore cannot go below 5–10 µm in diameter. In addition, during implantation, the devices are subjected to lateral forces, which explains why reported flexible brain interfaces typically use shuttle wires with diameters larger than the theoretical limit (e.g., 25 µm vs 5–10 µm).^[^ ^22^ ^]^ This may explain why the total surgical footprint of roughly 50 µm^2[^ ^112^ ^]^ has not been reproduced even when using the stiffest diamond materials.^[^ ^54^ ^]^ The acute tissue damage caused by the volume of the shuttle device becomes the most prominent source of injury for probe designs that come closest to our goals. While the acute butcher number approaches one in these cases, it still falls far from the few percent targeted. This precludes covering the entire tissue volume with electrodes, even though the shuttle devices are later retracted and the acute wounds heal over time.

An unconventional approach to deliver flexible electrodes into the tissue by magnetic acceleration was demonstrated in vivo.^[^ ^56^ ^]^ In these experiments, a 25 µm diameter, spear‐like shank with a ferromagnetic tip was injected into the tissue at high speed. While the authors reported functional single‐unit recordings over 30 days from rats, it is questionable whether the size of the projectile can be significantly reduced due to the magnetic forces being proportional to the volume of the device. Furthermore, high‐speed insertion (>20 m s^−1^) likely causes greater tissue damage than conventional insertion of shuttle wires, as in the latter cases, slow insertion speeds (<5 µm s^−1^) have been shown to improve the quality of acute neuronal recordings.^[^ ^73^ ^]^

Flexible neural probes are shown to induce less tissue reaction than rigid probes over chronic timescales. However, when we want to scale up measurements, we cannot afford a significant fraction of the tissue to be damaged by the shuttle devices during the implantation. Consequently, fundamental constraints related to occupied volume and bending stiffness limit the maximum achievable electrode density for flexible electrode probes, just as they do for self‐supporting rigid ones.

A further factor hindering successful network interfacing with both self‐supporting and flexible probes is internal bleeding caused by implantation, which is known to severely alter neural circuitry.^[^ ^67^, ^95^ ^]^ This issue is often overlooked, as many studies focus on the chronic effects of implants when bleeding and the associated inflammatory reactions have been recovered. Implantation technologies have been developed to minimize damage to the larger blood vessels by visualizing and adapting to shallow vessels during the procedure^[^ ^55^, ^75^ ^]^ or by using force feedback to avoid piercing vessels even deep within the tissue.^[^ ^89^ ^]^ When aiming for scaling up the technology, however, harming even small capillaries becomes intolerable because the large number of occurrences amplifies the effects of minor internal bleeding sites. At the same time, some degree of damage to the capillary network appears unavoidable, given the vasculature of the brain is dense and lacks straight, clear paths even for the insertion of microfibers.^[^ ^54^, ^75^, ^113^ ^]^ Implanting probes at slow speeds (<2 µm s^−1^) may help preserve the integrity of smaller capillaries^[^ ^73^ ^]^ and reduce long‐term astrocytic scarring.^[^ ^114^ ^]^ Additionally, some argue that atomically flat, smooth shank surfaces may also be beneficial in this respect.^[^ ^74^ ^]^ However, further studies are needed to assess the relevance of these effects in reducing acute tissue damage when a large number of micrometer‐sized probes are implanted.

Although we came close to achieving our goal of full‐volume recording, we fell short due to the fundamental physical limitations of tissue penetration. In the next section, we explore recent approaches that could potentially bypass the need for penetrating tissue with prefabricated devices.

## 5 Biohybrid Approaches

Biohybrid devices, which incorporate both artificial and living cellular elements, are often regarded as a promising future approach for neural interfaces. Various arrangements have been described, targeting multiple host tissues and leveraging different levels of integration of seeded cells into the signal transmission process.^[^ ^115^, ^116^ ^]^

Multiple research laboratories are focusing on improving biocompatibility in vivo by using cells seeded on probes, where the cells do not establish active connections outside the probe. This approach has led to more biocompatible neuron‐probe interfaces, with soft scaffolds, often hydrogels, reducing nervous injury during implantation and subsequent tissue response at the probe surface.^[^ ^80^, ^117^, ^118^ ^]^ However, these technologies are primarily intended to wrap rigid implants larger than 100 µm in diameter, which inevitably renders the final construct too large to be inserted into the cortex in large numbers.

The direct formation of active synapses between artificial elements and targeted neurons has been investigated by multiple research groups.^[^ ^119^ ^]^ For example, eliciting the formation of presynaptic terminals by neuroligin‐1‐functionalized probes has been reliably achieved with the aim of interfacing with neural circuitry.^[^ ^120^ ^]^ However, in this in vitro study, the postsynaptic sensing apparatus has not yet been developed. Meanwhile, numerous groups have developed miniaturized sensors for detecting neurotransmitters, as reviewed in various papers.^[^ ^119^, ^121^, ^122^ ^]^ Despite these advancements, none of these technologies have been demonstrated to interact with synaptic connections in vivo.

The neurotrophic electrode represented a groundbreaking approach, designed to record neural signals by promoting the growth of neural tissue onto the electrode surface.^[^ ^123^ ^]^ This was achieved through a specialized “cone electrode” that encouraged neurite growth using a neurotrophic factor, enabling stable long‐term recordings.^[^ ^124^ ^]^ Initially, the procedure garnered considerable attention as the probe allowed a patient to control a computer directly through thought, even years after implantation.^[^ ^125^ ^]^ Despite successes, the technology showed inconsistent performance across patients, in part due to variability in how well the brain tissue grew onto the electrodes. In the end, the project failed to meet clinical and technology development requirements, and the FDA withdrew approval for future procedures. While the probe's complexity, limited scalability of signal acquisition, and invasive nature posed significant challenges, the technology demonstrated the feasibility of a biohybrid brain interface.

**Figure 8:**
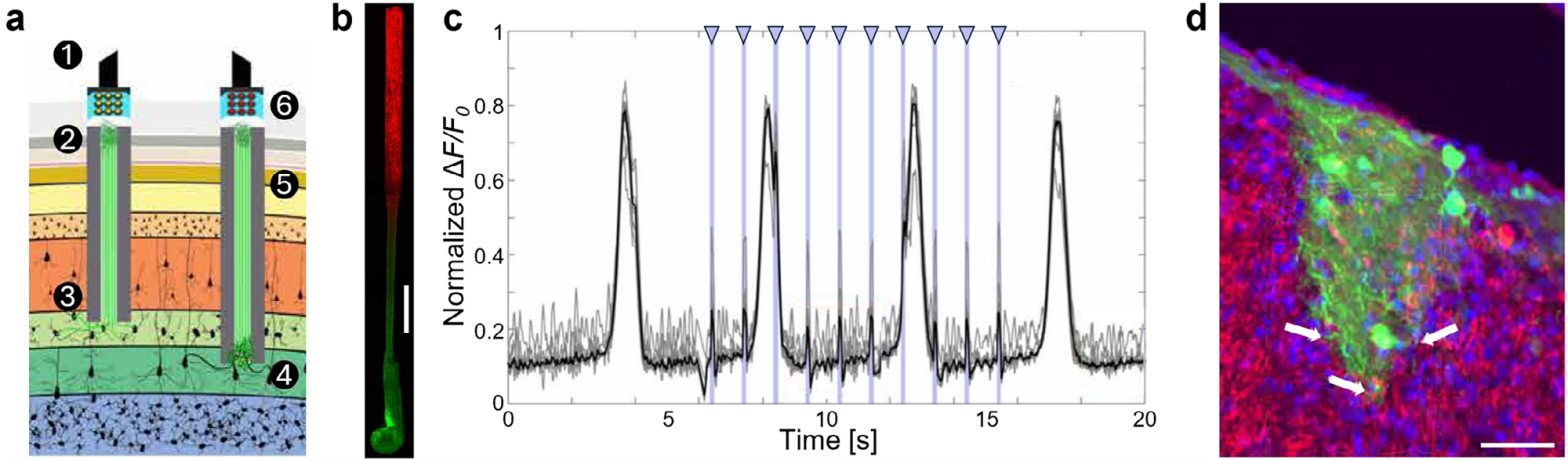
Living electrodes for brain interface. a) Concept of living electrodes for transplantable input/output channels. Inputs: A light‐emitting diode (LED) array 1) optically stimulates a unidirectional, channelrhodopsin‐positive µTENN 2) to activate layer IV neurons 3). Outputs: Layer V neurons 4) synapse with a bidirectional µTENN 5); relayed neuronal activity is recorded by a photodiode array on the brain surface 6). b) µTENN in vitro, virally transduced such that one aggregate expresses ChR2 optical actuator (bottom), and the other aggregate expresses the calcium reporter RCaMP (top), enabling simultaneous control and monitoring with light. Scale bar, 100 µm. c) Normalized pixel intensity of regions of interest within the RCaMP^+^ aggregate during stimulation in vitro. A train of 1‐Hz, 100‐ms stimulation pulses is shown as blue bands marked with triangles. Changes in pixel intensity due to stimulation of the input aggregate can be seen as sharp spikes occurring within the endogenous, large‐amplitude slow‐wave activity. d) Longitudinal view of implanted µTENN within the corticothalamic tract at 1 month after implantation, showing evidence that the construct retained its axonal tracts and overall axosomatic architecture in vivo. µTENN neurons and axons (GFP, green) were found projecting ventrally, with neurons and neurites interfacing with host tissue at the deep end. Red: NF200 (neurons/axons), blue: Hoechst (cell nuclei), purple: synapsin (synapses). Arrows denote µTENN neurites penetrating the host brain and putative synapse formation. Scale bar, 50 µm. Adapted under terms of the CC‐BY license.^[^ ^127^ ^]^ Copyright 2021, The Authors, published by AAAS.

A promising new approach for our purpose is the use of tissue‐engineered network elements (µTENN).^[^ ^126^ ^]^ In this concept, engineered neurons are implanted in the brain within a tube that later forms functional connections with the nearby tissue. These implanted neurons would detect network activity and activate other implanted neurons at the brain surface which would then be easily interfaced with electrocorticography (ECoG) devices or optical technologies (**Figure** **8**a). The beauty of the “living electrode” concept is that the implant's processes might form far‐reaching connections within the tissue without occupying significant volume or causing substantial damage, allowing a limited number of µTENN devices to sample an extensive brain circuitry. This architecture is capable of bidirectional information transmission in vitro and has been successfully sustained and tolerated by mouse subjects that were implanted with the devices (Figure 8b,c).^[^ ^127^ ^]^ Unfortunately, in the studies conducted so far, the connections between the implant and brain tissue have not been established in an organized way (Figure 8d), and, accordingly, the activity of cortical neurons has not been specifically detected. Moreover, these studies have not addressed how synapse formation could be controlled between the µTENN and the host tissue, how to achieve some kind of activity mapping, or what mechanisms drive and later stabilize the growth of the engineered network elements both within and outside of the transplant.

Multiple recent studies have advanced the applicability of biohybrid brain interfaces for the stimulation of neural activity. Pregrown fiber tracts have been implanted into the visual cortex of living mice with the goal of restoring vision.^[^ ^128^ ^]^ In this study, neural spheroids were electrically stimulated, and their fibers were guided into the brain using micromachined polymer grooves. While signal transmission via the processes has been demonstrated, no functional connections with the host tissue have been shown. Another group developed a 2D scaffold composed of microwells to house optogenetically enabled neurons individually. Upon implantation onto the brain surface, these neurons survived over several weeks and established connections with the host brain.^[^ ^129^ ^]^ Mice with these biohybrid implants were trained to perform tasks in response to optical stimulation, effectively demonstrating signaling through the neural grafts. These studies demonstrate substantial investment in the field, yet admittedly they do not address the control and guidance of synapse formation, without which forming large‐scale connections cannot be expected.

In summary, efforts to develop scalable biohybrid neural interfaces have demonstrated significant progress. However, these advancements also underscore the crucial importance of gaining a comprehensive understanding of the mechanisms governing tissue organization, the principles of synaptic targeting, and the fundamental communication and genetic processes involved. Deeper insight into these areas is essential for overcoming current challenges and fully realizing the potential of biohybrid neural interfaces.

## 6 Conclusion and Outlook

We discussed the need for a large number of electrodes to comprehensively sample neural activity and reviewed potential methods in light of recent literature. We assessed the limitations of rigid, self‐supporting probes (e.g., volume occupation^[^ ^26^ ^]^ and buckling resistance^[^ ^85^ ^]^) and found that even their most promising arrangements (e.g., rigid, ≈5 µm diameter shanks) are likely to cause significant FBR and tissue damage^[^ ^32^ ^]^ in large‐scale implants. In conjunction with this, when inserting many shanks, the dense vasculature makes internal bleeding unavoidable and triggers tissue reorganization, as there is no straight path through the tissue that does not intersect with at least some capillaries.^[^ ^113^ ^]^ Flexible electrodes can be smaller in cross‐section, but scalable applications must also account for the tissue damage caused by the insertion of implantation devices. The size reduction of shuttle devices is again limited by the buckling, and thus, the butcher number does not fall below 1 in these attempts. Although flexible electrodes could theoretically meander between capillaries, rigid shuttle devices lack this capability. To date, only solutions aimed at protecting larger blood vessels have been developed.^[^ ^55^, ^89^ ^]^ Consequently, the use of any prefabricated penetrating probes, whether self‐supporting or flexible, encounters fundamental physical limitations when aiming for whole‐brain sampling.

These electrodes could, in theory, be replaced by bioengineered cell processes and connections that may grow smoothly into the neural tissue. However, only some of the necessary aspects have been thoroughly investigated to date, and our current bioengineering knowledge is inadequate to address several challenges, including guiding the growth of neural processes and the formation of their connections, designing their topographical organization, and ensuring the long‐term stability of the implant over chronic time scales. Despite tremendous research efforts in these areas, fully understanding the genetic, cellular, and network mechanisms required for these features is still likely to be several decades away from becoming a practical engineering reality.

We introduced the acute butcher number as a simplified, conceptual metric for evaluating the scalability of various approaches in terms of the damage caused at the electrode–tissue interface. To enable recording from a large fraction of neurons with minimal perturbation, it is a minimum requirement that this value converges to zero or, at most, remains within a few percent. Our review identifies carbon fiber electrodes and flexible thread electrodes implanted via shuttle wires as particularly promising candidates for scaling up. However, even in these advanced cases, the acute butcher number remains above one, indicating that substantial further progress is needed to overcome inherent challenges associated with the physical constraints of brain tissue and device mechanics.

In our view, the following guidelines could serve as key milestones toward achieving this goal: i) An ideal device should not require implantation by pressing rigid bodies into the tissue; instead, micrometer‐scale fibers should enter the tissue through some kind of growth method. ii) The growth, orientation, and distribution of these fibers must be delicately controlled to ensure that the readout properly samples the neural network architecture of the brain. iii) The thin, flexible processes/electrodes formed should not trigger FBR over chronic timescales. In conjunction with this, the material composition of the device should not activate glial cells or the immune system in general. iv) During formation, the fibers or processes should avoid colliding with or piercing cellular elements, preserving the integrity of nearby neurons and capillaries.

Although these criteria may seem unattainable today, they can be pursued through at least two significantly different approaches. The first approach is to leverage the latest advances in materials science to grow such fine structures directly within the neuronal tissue. For example, we may construct electrodes within the neuronal tissue through local polymerization,^[^ ^130^ ^]^ complemented by precise control over conductive properties at the micrometer scale to create a conductive core and an insulating outer layer. The second approach is to advance the knowledge of synthetic biology in creating new synaptic connections with the brain. This could involve using engineered cells that autonomously grow their neurites into the desired cortical layer and form efferent connections with the neurons. Transplanted neuronal cells are likely to integrate into the tissue according to their genetic program, a process that has been extensively studied with the goal of repairing damaged brain tissue.^[^ ^131^ ^]^ Building on these results, modified cells could transfer information to an easily accessible area of the brain, such as the cortical surface, where it could be interfaced with electronic devices.

These avenues appear challenging and face numerous obstacles, but through such a bio‐integrative paradigm shift, we may overcome the fundamental limitations of current technology and realize the ambitious goal of recording the electrical activity of most cortical neurons.

## Conflict of Interest

The authors declare no conflict of interest.
